# Mindfulness and Self-Compassion in Generalized Anxiety Disorder: Examining Predictors of Disability

**DOI:** 10.1155/2013/576258

**Published:** 2013-09-23

**Authors:** Elizabeth A. Hoge, Britta K. Hölzel, Luana Marques, Christina A. Metcalf, Narayan Brach, Sara W. Lazar, Naomi M. Simon

**Affiliations:** ^1^Department of Psychiatry, Massachusetts General Hospital, Boston, MA, USA; ^2^Center for Anxiety and Traumatic Stress Disorders, Massachusetts General Hospital, One Bowdoin Square, 6th Floor, Boston, MA 02114, USA; ^3^Charite University Medicine, Institute of Medical Psychology, Berlin, Germany

## Abstract

Generalized anxiety disorder (GAD) is a condition characterized by worry and physiological arousal symptoms that causes significant disabilities in patients' lives. In order to improve psychotherapeutic interventions, a careful characterization of the deficiencies of this population as well as factors that ameliorate disability is crucial. Variables that have not traditionally been the focus of research should be considered, such as trait mindfulness and self-compassion. We investigated whether GAD patients would report lower mindfulness and self-compassion levels than healthy stressed individuals. Eighty-seven GAD patients and 49 healthy controls completed the Five Facet Mindfulness Questionnaire, the Self-Compassion Scale, and measures of anxiety. Patients with GAD also completed the Sheehan Disability Scale. Results showed that GAD patients had lower mindfulness and self-compassion than healthy stressed controls, and both were negatively correlated with levels of anxiety, worry, and anxiety sensitivity. In patients, mindfulness was a better predictor of disability than actual anxiety symptom scores. These findings highlight that in the presence of anxiety symptoms, mindfulness can be a factor that helps protect against feeling disabled by the disorder. The findings thereby add an important variable to the characterization of this disorder and should be taken into consideration for future treatment development.

## 1. Introduction

With a lifetime prevalence of approximately 5.7% (National Comorbidity Survey Replication, [1]), generalized anxiety disorder (GAD) is one of the most common anxiety disorders. GAD patients suffer from excessive worry and physiological arousal symptoms such as poor sleep, muscle tension, and irritability. The disorder causes significant disabilities in patients' lives; for example, GAD is associated with significant impairment in social and general functioning [2]. According to a WHO study, 38% of individuals with GAD had moderate to severe occupational role impairment, with a mean of 6.3 disability days per month [3]. Furthermore, GAD is also associated with both increased health care utilization [4] and comorbid health problems. Disability is, thus, a significant problem in GAD. 

In order to advance treatment approaches, it is crucial to carefully describe the characteristics of the patient population and determine which factors are protective against the social and occupational disability that is associated with the disorder. With newer self-report instruments, further characterization of GAD patients has become possible. Two traits that have recently received a lot of attention in the context of mindfulness-based therapeutic approaches are “mindfulness” and “self-compassion.” 

Mindfulness is typically defined as nonjudgmental attention to experiences in the present moment [5, 6]. It has been well established that mindfulness contributes to mental health [7, 8]. There is very little empirical data to demonstrate that mindfulness levels are low in GAD patients. So far, only one small study has reported that levels of mindfulness are significantly lower in GAD patients compared to nonanxious controls (*N* = 16, [9]). 

Self-compassion has been defined as being “open to and moved by one's own suffering, experiencing feelings of caring and kindness towards oneself, taking an understanding, nonjudgmental attitude towards one's inadequacies and failures, and recognizing that one's own experience is part of the common human experience” [10]. This emerging psychological construct has been shown to be positively associated with measures of psychological health, such as life satisfaction, social connectedness, and emotional intelligence [11], and commitment to making adaptive health changes in one's life [10, 12]. Aside from lower mindfulness in the small sample of GAD patients, Roemer and colleagues have also reported lower scores in self-compassion compared to nonanxious individuals [9]. In a large community sample of treatment-seeking but nondiagnosed anxious individuals, self-compassion was associated with symptom severity and quality of life [13].

In their current operationalizations [10, 14] mindfulness and self-compassion are highly correlated, and a correlation coefficient of *r* = 0.69 has been reported in a nonclinical population [15]. In the large sample of nondiagnosed anxious individuals, self-compassion accounted for ten times more unique variance in the dependent variables than mindfulness [13].

These data suggest that self-compassion and mindfulness are low in anxiety disorders. However, studies are limited by small sizes [9] and lack of clinical diagnoses [13], and therefore the contribution of both variables to suffering in GAD has not entirely been determined. Furthermore, both of these studies have employed the Mindful Attention Awareness scale (MAAS, [7]), which is operationalized to mostly address the attentional component of mindfulness. The five facet mindfulness questionnaire (FFMQ, [14]) allows for a more complex assessment of various aspects of the mindfulness construct and how they might contribute to the suffering in GAD. 

Both mindfulness and self-compassion are constructs that describe how individuals relate to themselves and to their experiences, including unpleasant experiences such as pain and fear. Pain research demonstrates that mindfulness interventions can help patients with chronic pain to enhance well being and quality of life, even when chronic pain symptoms continue [16, 17]. In this context, a distinction is often made between “pain” versus “suffering” (c.f. [18]). While pain refers to the sensory aspect of the experience, suffering refers to the subjective emotional experience that emerges from an internal resistance against a specific experience [19]. Mindfulness research on pain reveals that with mindfulness training, an experience is possible where while painful sensations might be experienced, and patients might not necessarily need to suffer from them. Rather, pain sensations can be mindfully experienced as what they really are, namely, sensory experiences. While this line of argumentation stems from pain research, the principle can be easily transferred to the field of anxiety. Here, the idea is that anxiety symptoms can be experienced, but with increased mindfulness and self-compassion, patients might feel less disabled by their disorder. It is, therefore, possible that mindfulness and self-compassion might ameliorate the amount of disability an individual experiences from their disorder, even in the presence of high levels of symptoms. Data supporting this role for self-compassion come from research by Neff and McGehee [20], who found that self-compassion was linked to resilience in adolescents and young adults. No previous studies, however, have investigated the impact of mindfulness or self-compassion on disability in GAD.

Understanding the contribution of low mindfulness and self-compassion in GAD is of potential clinical relevance, as their cultivation may be a potential under-addressed treatment target in patients with anxiety. In the current study, we aimed to replicate and extend previous findings by examining the levels of mindfulness and self-compassion in individuals with GAD compared to healthy individuals experiencing life stress and their relationship with disability. We hypothesized that mindfulness and self-compassion would be lower among GAD patients than healthy stressed subjects and would be associated with greater disability, which would provide support for their potential consideration as treatment targets.

## 2. Materials and Methods

### 2.1. Participants

Individuals with current GAD as defined by the DSM-IV-TR [21] criteria and healthy controls with high ratings of subjective stress were recruited to the Massachusetts General Hospital Department of Psychiatry to participate in a stress reduction course. The patients and controls were recruited through two separate protocols by different staff, during the same time period (2008 to 2011).

All participants underwent a psychiatric diagnostic assessment that was performed by trained M.D. or Ph.D. level study clinicians using the Structured Clinical Interview for DSM-IV [22]. Patients with anxiety were eligible to participate if they were outpatients with a primary psychiatric diagnosis of generalized anxiety disorder (GAD) and above 18 years of age. Additionally, patients must have received a rating of 16 or higher on the HAM-A [23] during screening to be eligible, as this score of symptom severity generally indicates moderate GAD [24, 25].

Stressed healthy controls were eligible if they did not have any current psychiatric disorder and did not meet a diagnosis within the last six months. Furthermore, they were eligible if their score on the four-item Perceived Stress Scale (PSS) was higher than the population mean of 4.5 [26, 27], if they were right handed, and between the ages of 23–60. History of significant head trauma, presence of metallic body implants, or a history of claustrophobia was exclusionary, as the protocol with stressed healthy subjects had a neuroimaging component. 

Eligibility for both groups required that participants be medically healthy and free from a lifetime history of schizophrenia or any other psychosis, mental retardation, organic medical disorders, bipolar disorder, posttraumatic stress disorder, or obsessive compulsive disorder (OCD), and alcohol or substance abuse or dependence within the past 6 months. In the GAD group, patients taking antidepressants or benzodiazepines were allowed by other psychiatric medications, such as antipsychotics and anticonvulsants were exclusionary. Psychiatric medications of any kind were exclusionary for the stressed healthy controls. 

Exclusion criteria for both groups included significant previous meditation or yoga experience. Meditation experience extending beyond four meditation classes in the past five years or more than ten classes more than five years ago was exclusionary; yoga experience in excess of eight classes within the past year, more than eight consecutive weekly classes within the past five years, or more than sixteen consecutive weekly classes more than five years ago was exclusionary.

### 2.2. Procedure

Written consent was obtained from all participants and the study protocols were approved by the MGH institutional review board. Participants received diagnostic screens using the Structured Interview for DSM-IV-TR Axis I Disorders [21] and filled out self-report questionnaires.

### 2.3. Measures

#### 2.3.1. Measures for All Subjects

*(1) Self-Compassion Scale (SCS)*. The SCS is a 26-item self-report instrument that uses a 5-point Likert scale for each item to assess compassion and kindness towards oneself, partially through seeing one's experiences as not unique and specific to the individual but part of a larger human experience [28]. Individuals respond to how often they behave in the manner that is described in each statement, from “almost never” (1) to “almost always” (5). Examples include, “I try to be loving towards myself when I'm feeling emotional pain,” and “when things are going badly for me, I see the difficulties as part of life that everyone goes through.” The SCS has six subscales. The positive subscales are self-kindness, common humanity (realizing that one's experiences are similar to others), and Mindfulness, and three negative subscales which are thought to reflect the same concepts but in the negative state, which are reverse-coded; and self-judgment, isolation, and over-identification (becoming absorbed in one's situation and emotional reaction, losing a more objective perspective), respectively. Scores on the subscales are averaged to compute the total SCS score, with a range of possible total scores from 1.00 to 5.00; this is a change from the summary score method to the mean score calculation method, as proposed by the author's scale [29]. The SCS has demonstrated good reliability and validity in English, Thai, and Taiwanese [10, 30].

*(2) Five Facet Mindfulness Questionnaire (FFMQ)*. The Five Facet Mindfulness Questionnaire (FFMQ, [14]) is a 39-item scale that was developed based on an item pool of previously existing mindfulness questionnaires. Factor analyses over these items yielded five facets of mindfulness: observing (attending to or noticing internal and external stimuli such as sensations, emotions, cognitions, sights, sounds, and smells); describing (noting or mentally labeling these stimuli with words); acting with awareness (attending to one's current actions, as opposed to behaving automatically or absent-mindedly); nonjudging of inner experience (refraining from evaluation of one's sensations, cognitions, and emotions); and nonreactivity to inner experience (allowing thoughts and feelings to come and go, without attention getting caught in them). Responses to the items are given on a 5-point Likert-type scale (1 = never or very rarely true, 5 = very often or always true). The five subscales have presented with adequate to good internal consistency [14].

*(3) The Structured Clinical Interview for DSM-IV (SCID)*. The SCID is a semistructured interview for making the major Axis I DSM-IV diagnoses such as anxiety and affective disorders, alcohol and substance abuse and dependence, eating disorders, and psychotic disorders [22].

*(4) Anxiety Sensitivity Index (ASI)*. The ASI is a 16-item self-report instrument designed to assess fear of anxiety and to measure individuals' discomfort with a variety of sensations associated with anxiety and panic [31]. Individuals score items on a 5-point Likert scale.

*(5) Penn State Worry Questionnaire (PSWQ)*. The PSWQ is a 16-item self-rated measure that is used to measure the generality, excessiveness, and uncontrollability of pathological worry [32]. Each item is scored on a 5-point Likert scale and summed to form a total score ranging from 16 to 80. Individuals with GAD have been shown to score significantly higher on the PSWQ than individuals with other anxiety disorders [33]. The PSWQ has excellent psychometric properties in student, community, and clinical samples [34].

*(6) State Trait Anxiety Inventory Trait (STAI)*. The STAI is a standardized questionnaire for assessing anxiety; the trait anxiety subscale includes 20 items that evaluate a more general and long-standing quality of “trait anxiety” [35]. Respondents indicate how they generally feel about each statement on a 4-point Likert scale, from “almost never” (1) to “almost always” (4). 

#### 2.3.2. Measures for GAD Individuals Only

*(1) Sheehan Disability Scale (SDS)*. The SDS is a 3-item measure of self-reported social and work related disability due to psychological symptoms [36]. The patient rates the extent to which life is impaired by his or her symptoms on a 10-point-visual analog scale, which uses spatio visual, numeric, and verbal descriptive anchors simultaneously to assess disability. The three items are in the form of “the symptoms have disrupted your … (work/school work; social life/leisure activities; and family life/home responsibilities).”

*(2) Beck Anxiety Scale (BAI)*. The BAI is a 21-item questionnaire measuring severity of anxiety symptoms, including nervousness, fear, and somatic symptoms, in psychiatric populations, shown to have high internal consistency (0.90 to 0.94) and test-retest reliability (0.67 to 0.93); [37].

#### 2.3.3. Measures for Healthy Controls Experiencing Stress Only

*(1) Perceived Stress Scale (PSS; [27])*. The PSS is a validated self-report questionnaire widely used for assessing an individual's self-perception of stress. The PSS has 14-, 10-, and 4-item versions and has been shown to yield adequate reliability and validity [26, 27]. This study used the 4-item version as an inclusion measure for controls. The scale inquires about feelings and thoughts over the past month, and respondents indicate how often they felt a certain way using a 5-point-Likert scale (0 = never, 4 = very often); responses are then summed. Normative scores have been found to be 4.2 (SD 2.8) and 4.7 (SD 3.1), for men and women, respectively. 

### 2.4. Statistical Methods

Demographic and clinical differences between the GAD group and controls were examined using independent samples *t*-tests and Chi-square tests for categorical variables. Associations between clinical variables were determined by running correlation analyses. Multiple regression analyses were performed using anxiety and self-compassion as predictors of disability in the GAD sample. The BAI was used as a measure of anxiety symptoms, as previous work has demonstrated that the BAI score reflects overall anxiety symptom severity [38–40]. Statistical significance was set at *P* = 0.05.

## 3. Results

### 3.1. Sample

The sample consisted of 87 individuals meeting DSM-IV criteria for current GAD (51.22% females; mean age 39.4 years; SD 13.0 years) and 49 healthy controls with high ratings of subjective stress but no psychiatric diagnoses (65.31% females; mean age 38.7 years; SD 10.9 years). Of the GAD patients, 31.25% (and 0% of the healthy controls) were taking psychiatric medications. There were no significant statistical differences in age, gender, and race between the two groups, and thus these variables were not entered as covariates in the main analysis (see Table 1).

### 3.2. Clinical Anxiety, Mindfulness, and Self-Compassion Measures

As hypothesized, patients with GAD had significantly lower scores on the SCS than did the controls. Patients with GAD had a mean (SD) score of 2.44 (0.59) on the total SCS, compared to 3.17 (0.60) in the healthy controls (*t* = 6.89, *P* < 0.001). All of the subscales were also significantly different, with the GAD patients scoring lower in self-kindness, common humanity, and mindfulness, and higher in self-judgment, isolation, and over-identification (all *P*s < 0.003, see Table 1). GAD patients also had lower scores on the FFMQ scales acting with awareness, nonjudging, and nonreactivity (all *P*s < 0.001, see Table 1), compared to healthy participants. Scores were not statistically different on the scales describing (*P* = 0.43) and observing (*P* = 0.07). FFMQ and SCS total scores were highly correlated in GAD patients (*r* = 0.62, *P* < 0.001) and healthy participants (*r* = 0.77, *P* < 0.001). Consistent with our hypothesis, patients with GAD also had significantly higher scores on the PSWQ (*P* < 0.001), STAI (*P* < 0.001), and ASI (*P* < 0.001) than did controls (see Table 1).

There was no difference in self-compassion scores between men and women, neither in the sample of GAD patients (all *P*s > 0.14), nor in the healthy participants (all *P*s > 0.117). There were also no gender differences in FFMQ scores in GAD patients (all *P*s > 0.07) or healthy participants (all *P*s > 0.13). 

Further, in the whole sample, as well as in both GAD patients and healthy participants separately, both SCS and FFMQ scores were negatively correlated with trait anxiety, worry, and anxiety sensitivity (see Table 2). All correlations, except for the correlation between FFMQ and ASI scores in healthy participants were significant. 

### 3.3. Self-Compassion, Mindfulness and Anxiety as Predictors for Disability in GAD

Fourteen of the 87 GAD patients were missing BAI scores, as this scale was introduced to the study four weeks after the study began. In order to assess the relevance of lower mindfulness and self-compassion scores for disability in GAD patients, regression analyses were performed to predict levels of the Sheehan Disability Scale. Since anxiety symptoms are an obvious predictor for disability in GAD patients, BAI scores were also used as a further predictor. When considering self-compassion, FFMQ mindfulness and anxiety severity (as measured by the BAI) individually, all three were significantly correlated with disability (SCS: *r* = −0.38, *P* < 0.001, FFMQ: *r* = −0.512, *P* < 0.001, and BAI: *r* = 0.29, *P* = 0.014, resp.). 

 When entering all three variables simultaneously into a multiple regression, only the FFMQ total score (*B* = −0.17, SE = 0.06, *P* = 0.003) but not anxiety symptom severity on the BAI (*B* = 0.15, SE = 0.09, *P* = 0.113) or SCS (*B* = −0.90, SE = 1.62, *P* = 0.581) was significantly associated with greater functional disability (*F*(3,69) = 8.41, *P* < 0.001). This association was still significant when medication use was added as a covariate (*P* = 0.002).

To determine what aspects of mindfulness were most strongly related to disability, we ran an additional analysis, entering each of the five subscales into the multiple regression. The factors nonjudging (*P* = 0.010) and describing (*P* = 0.026) significantly predicted disability.

## 4. Discussion

The current study found that individuals with GAD reported lower mindfulness and self-compassion than controls, and that both were negatively correlated with levels of anxiety, worry, and anxiety sensitivity. As expected, GAD patients had higher levels of worry and anxiety overall. Intriguingly, mindfulness was associated with levels of disability above and beyond the effects of anxiety symptom severity in GAD. 

The findings of lower mindfulness and self-compassion levels in GAD corroborate earlier research [9]. Our findings were also similar to other research in anxiety disorders. Werner and colleagues demonstrated that patients with social anxiety disorder, a disorder also associated with ruminative worry but which is focused on social interactions, have lower self-compassion than controls [41]. Taken together with our study, this may suggest that anxiety of several types might be associated with low mindfulness and self-compassion.

The data presented here provide additional evidence regarding the unique contributions of mindfulness (and secondarily also of self-compassion) to disability. The finding that the level of mindfulness is a stronger predictor than anxiety symptoms per se in the prediction of disability emphasize the importance of considering mindfulness as a crucial variable in the description of GAD and when tailoring behavioral interventions to treat GAD. The usefulness of mindfulness-based interventions for the amelioration of anxiety symptoms has been confirmed by a recent meta-analysis [42]. 

Our study results suggest that low trait mindfulness is related to psychiatric disability, beyond the effect of anxiety on disability, which reveals another pathway to disability potentially not sufficiently affected by current treatments. This finding is similar to research by Wren and colleagues, who demonstrated that among patients with chronic pain, low self-compassion was associated with higher pain disability [43]. The authors speculate that higher self-compassion might enable patients to “have an accepting attitude toward their day-to-day limitations without ignoring or fixating on them,” leading them to also accept emotions related to pain disability, “while still maintaining engagement in meaningful day-to-day activities.” 

Our findings demonstrate that it is particularly higher levels of nonjudging of inner experience (refraining from evaluation of one's sensations, cognitions, and emotions) and describing (noting or mentally labeling these stimuli with words) that contribute to lower disability above and beyond anxiety symptom severity, suggesting that they are protective in the presence of anxiety symptoms. The ability to label one's emotions without evaluating them might be an important skill for patients, which might help them continue to pursue meaningful activities despite the presence of anxiety and promote better overall functioning. Labeling one's affect has been suggested to be an incidental emotion regulation process that can attenuate distress [44]. Not judging ones experience, but noting it verbally, might lead to a state of decentering; that is, the capacity to observe phenomena as temporary, objective events in the mind, rather than reflections of the self that are necessarily true [45]. The data of this study, thus, corroborate the argument that the ability to mindfully be in contact with one's emotions can be protective of disability, despite the presence of anxiety symptoms.

The present findings are limited by the fact that subjects in the GAD study and in the healthy control study were recruited by two different research groups for two separate protocols, which may have resulted in bias. However, both groups received identical self-report questionnaires with the same instructions, during the same overall period of time and in the same institution, and both used clinician-administered diagnostic assessments to carefully determine diagnoses. Second, the study is limited by having only one measure of disability, rather than a combination of self-report and objective measures. Finally, it is important to emphasize that some would argue that the definition of “mindfulness” has not yet reached a consensus [46] nor does the concept of mindfulness yet have a consistent measuring instrument [47, 48]. However, the results of our study emphasize the usefulness of the FFMQ in characterizing GAD patients, and its important contribution to describe patients' disability. 

In conclusion, these data support that mindfulness and self-compassion are worthwhile constructs to further explore in GAD treatment research, not only because they may contribute to suffering in addition to that resulting from anxiety, but also because of the independent relationship of lower mindfulness with greater disability, which suggests it may be an important target for intervention. Future work should confirm the presence of low mindfulness and self-compassion in a single, large study, and examine how they are impacted by treatments that specifically target it, such as mindfulness meditation training, compared to those that do not, such as pharmacotherapy.

## Figures and Tables

**Table 1:** Demographic and clinical comparison of GAD and controls.

	GAD *N* = 87	Controls *N* = 49	*P* value
Age, Mean (SD)	39.4 (13.0)	38.7 (10.9)	0.76
Race, % white (*n*)	87.25% (89)	75.51% (37)	0.10
Gender, % female (*n*)	51.22% (42)	65.31% (32)	0.15
SCS self-kindness (SD)	2.47 (0.76)	2.94 (0.74)	0.001
SCS self-judgment (SD)	2.77 (0.77)	1.93 (0.82)	<0.001
SCS common humanity (SD)	2.61 (0.92)	3.11 (0.85)	0.002
SCS isolation (SD)	2.68 (0.83)	1.71 (0.93)	<0.001
SCS mindfulness (SD)	2.81 (0.86)	3.31 (0.67)	0.001
SCS over-identified (SD)	2.78 (0.76)	1.69 (0.85)	<0.001
SCS total (SD)	2.44 (0.59)	3.17 (0.60)	<0.001
STAI-T mean (SD)	54.72 (7.79)	40.97 (8.53)	<0.0001
PSWQ mean (SD)	65.51 (9.36)	47.24 (12.27)	<0.0001
ASI mean (SD)	27.61 (11.32)	18.02 (10.00)	<0.0001
FFMQ describing (SD)	25.88 (6.67)	26.79 (6.02)	0.43 ns
FFMQ observing (SD)	24.52 (6.39)	22.44 (6.27)	0.07 ns
FFMQ acting with awareness (SD)	22.21 (6.41)	26.73 (6.10)	<0.001
FFMQ nonjudging (SD)	22.38 (6.90)	31.27 (6.25)	<0.001
FFMQ nonreactivity (SD)	17.57 (4.24)	21.27 (5.47)	<0.001
FFMQ avg (SD)	22.51 (3.50)	25.70 (3.66)	<0.001

Note: *t*-tests used for continuous, and chi square for categorical variable.

**Table:** (a)

	Age	PSWQ	STAI	SCS	ASI
PSWQ	−0.16				
STAI	−0.14	0.79**			
SCS	0.16	−0.67**	−0.76**		
ASI	−0.09	0.50**	0.50**	−0.46**	
FFMQ	0.21*	−0.59***	−0.70***	0.74***	−0.45***

*P < 0.05, **P < 0.01, ***P < 0.001.

**Table:** (b)

	Age	PSWQ	STAI	SCS	ASI
PSWQ	−0.35***				
STAI	−0.33**	0.57***			
SCS	0.28**	−0.57***	−0.68***		
ASI	−0.16	0.30**	0.37***	−0.32**	
FFMQ	0.43***	−0.44***	−0.615***	0.62***	−0.39***

*P < 0.05, **P < 0.01, ***P < 0.001.

**Table:** (c)

	Age	PSWQ	STAI	SCS	ASI
PSWQ	−0.86				
STAI	0.07	0.74***			
SCS	0.04	−0.44**	−0.63***		
ASI	0.03	0.46**	0.32*	−0.33*	
FFMQ	−0.17	−0.48***	−0.63***	0.77***	−0.26

*P < 0.05, **P < 0.01, ***P < 0.001.
